# Fast‐forward genetics by radiation hybrids to saturate the locus regulating nuclear–cytoplasmic compatibility in *Triticum*

**DOI:** 10.1111/pbi.12532

**Published:** 2016-02-24

**Authors:** Filippo M. Bassi, Farhad Ghavami, Matthew J. Hayden, Yi Wang, Kerrie L. Forrest, Stephan Kong, Rhoderissa Dizon, Monika K. Michalak de Jimenez, Steven W. Meinhardt, Mohamed Mergoum, Yong Q. Gu, Shahryar F. Kianian

**Affiliations:** ^1^ Department of Plant SciencesNorth Dakota State UniversityFargoNDUSA; ^2^ International Center for the Agricultural Research in the Dry Areas (ICARDA)RabatMorocco; ^3^ Eurofins BioDiagnostics, Inc.River FallsWIUSA; ^4^ Department of Environment and Primary IndustriesAgriBiosciences CenterBundooraVicAustralia; ^5^ USDA‐ARSWestern Regional Research CenterAlbanyCAUSA; ^6^ Department of Plant PathologyNorth Dakota State UniversityFargoNDUSA; ^7^ USDA‐ARS Cereal Disease LaboratoryUniversity of MinnesotaSaint PaulMNUSA; ^8^ Present address: Department of Crop and Soilthe University of Georgia1109 Experiment StGriffinGA30223

**Keywords:** speciation, *species cytoplasm specific*, fast‐forward genetics, BulkSeq, synteny, wheat

## Abstract

The nuclear‐encoded *species cytoplasm specific* (*scs*) genes control nuclear–cytoplasmic compatibility in wheat (genus *Triticum*). Alloplasmic cells, which have nucleus and cytoplasm derived from different species, produce vigorous and vital organisms only when the correct version of *scs* is present in their nucleus. In this study, bulks of *in vivo* radiation hybrids segregating for the *scs* phenotype have been genotyped by sequencing with over 1.9 million markers. The high marker saturation obtained for a critical region of chromosome 1D allowed identification of 3318 reads that mapped in close proximity of the *scs*. A novel *in silico* approach was deployed to extend these short reads to sequences of up to 70 Kb in length and identify candidate open reading frames (ORFs). Markers were developed to anchor the short contigs containing ORFs to a radiation hybrid map of 650 individuals with resolution of 288 Kb. The region containing the *scs* locus was narrowed to a single Bacterial Artificial Chromosome (BAC) contig of *Aegilops tauschii*. Its sequencing and assembly by nano‐mapping allowed rapid identification of a *rhomboid* gene as the only ORF existing within the refined *scs* locus. Resequencing of this gene from multiple germplasm sources identified a single nucleotide mutation, which gives rise to a functional amino acid change. Gene expression characterization revealed that an active copy of this *rhomboid* exists on all homoeologous chromosomes of wheat, and depending on the specific cytoplasm each copy is preferentially expressed. Therefore, a new methodology was applied to unique genetic stocks to rapidly identify a strong candidate gene for the control of nuclear–cytoplasmic compatibility in wheat.

## Introduction

The nucleus and the organelles cross talk in a complex network of proteins and mRNA exchanges. Genes encoded in the nuclear and cytoplasmic genomes are responsible for governing this mechanism defined as nuclear–cytoplasmic interactions (NCI). This interaction is quintessential for the life of a plant cell and is ultimately involved in nearly all its molecular functions. Throughout evolution, specific combinations of nucleus and cytoplasm have been selected to optimize the fitness of a species (Michalak *et al*., 2013). When the compatibility between the nucleus and the cytoplasmic organelles is compromised by interspecies hybridizations, the results can be dramatic, often leading to premature death of the hybrid or its inability to procreate (Maan, 1991). In other instances, it has led to the evolution of entirely new species with a new NCI balance, as in the case of allotetraploid [(*T. turgidum* ssp *durum* L (2n = 4x = 28, AABB)] and allohexaploid wheat [*Triticum aestivum* ssp *aestivum* L. (2n = 6x = 42, AABBDD)] (Dvorak *et al*., 1993; Kilian *et al*., 2007; Matsuoka, 2011) and many other lineages of the *Triticeae* tribe such as *T. timopheevii* ssp *timopheevii* Zhuck (2n = 4x = 28, AAGG) (Kilian *et al*., 2007; Sarkar and Stebbins, 1956). Interestingly, tetraploid durum wheat, hexaploid bread wheat, and tetraploid *T. timopheevii* share a related maternal ancestor (*Aegilops speltoides* ssp.) (Kilian *et al*., 2007). Hence their nuclei should theoretically be immersed in nearly identical cytoplasm (S‐type plasmon). However, co‐evolution between the different nuclear genomes and their cytoplasmic counterparts resulted in two novel plasmons that differ from their maternal origin, plasmon B for wheat and plasmon G for *T. timopheevii* (Michalak *et al*., 2013; Noyszewski *et al*., 2014; Tsunewaki, 2009). In this complex phylogenetic scenario, it becomes apparent the evolutionary importance of the nuclear‐encoded *species cytoplasm specific* (*scs*) genes, which orchestrate the compatibility between the nucleus and the different plasmons.

Several *scs* genes have been identified on the group 1 chromosomes of *Triticeae* such as *scs* ^*ti*^ on chromosome 1A of *T. timopheevii*, and *scs* ^*ae*^ on chromosome 1D of *T. aestivum,* in addition to genes derived on telocentric chromosomes from other species (Hossain *et al*., 2004a; Maan, 1991; Maan and Kianian, 2002a,b). Homoeoallelic forms of these genes have also been identified on chromosome 1A of durum (Gehlhar *et al*., 2005), 1G of *T. timopheevii* (Asakura *et al*., 2000), 1D of *A. tauschii* Coss (syn *T. tauschii*,* A. squarrosa*; 2n = 2x = 14, DD) **(**Ohtsuka, 1980), and even 1H of *Hordeum vulgaris* L. (2n = 2x = 14, HH) (Taketa *et al*., 2002). The detailed analyses that led to the identification of the various forms of *scs* genes were made possible in wheat by the existence of cytoplasmic substitution lines. These lines have the nucleus of one species immersed in the cytoplasm of a different species (alloplasmic lines: *allo*‐alien *plasmon*‐cytoplasm). The disruption in alloplasmic conditions of the ‘wild‐type’ NCI has made it possible to investigate new forms of compatible interactions and to identify the nuclear and cytoplasmic components that regulate them.

**Figure 1:**
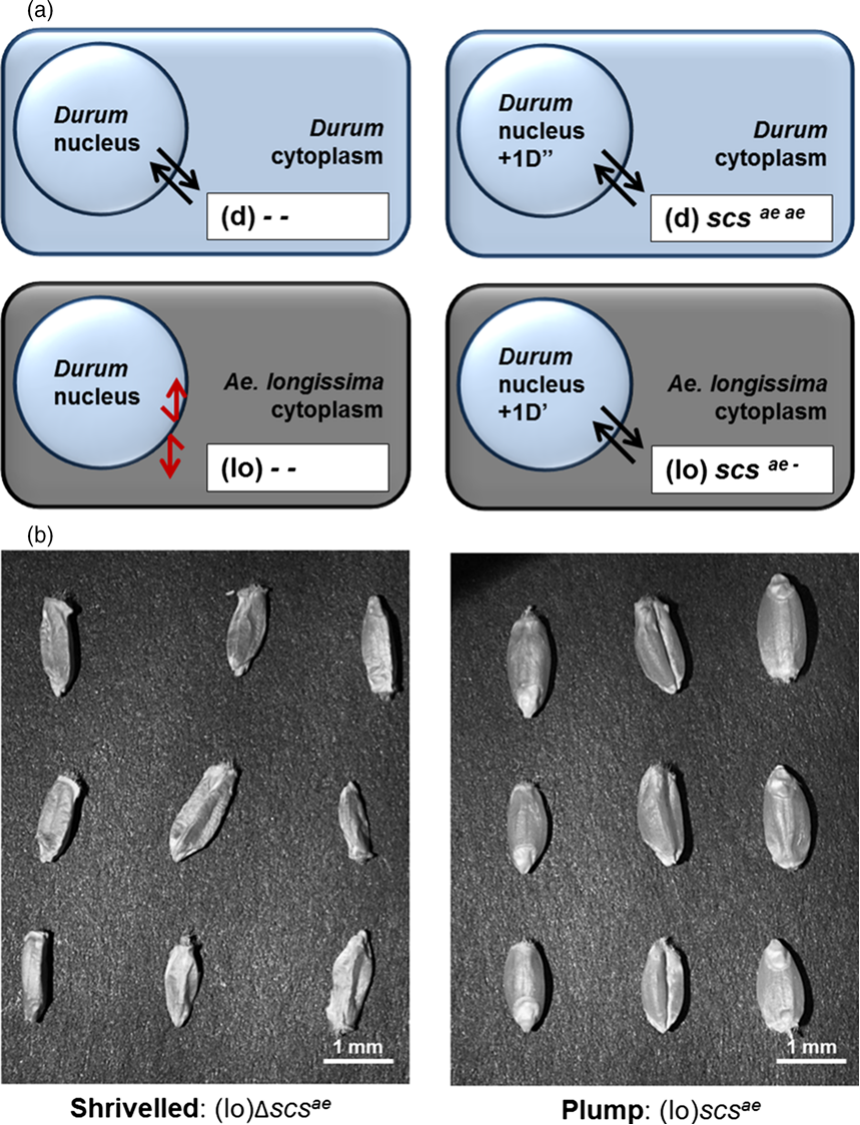
The mode of action of the *scs* ^*ae*^ gene in alloplasmic lines of wheat. (a) The black parallel arrows indicate compatible interaction between the nucleus and the cytoplasm, while the red broken arrows indicate incompatible interaction. In euplasmic lines with the nucleus of *T. durum* (d) and cytoplasm of the same species (d), the *scs* ^*ae*^ gene is not needed. In alloplasmic lines with the nucleus of *T. durum* (d) and cytoplasm of *A. longissima* (lo), the *scs* ^*ae*^ gene controls the correct nuclear–cytoplasmic interaction and one copy is sufficient to re‐establish seeds’ vitality. The absence of *scs* ^*ae*^ is shown as ‘–’ symbol, and the complete lack of *scs* ^*ae*^ in alloplasmic lines results in nonvital interaction (lo) ‐ ‐. (b) The alloplasmic mutants missing the *scs* ^*ae*^ gene [∆*scs* ^*ae*^ or (lo) ‐ ‐] have shrivelled seeds that do not germinate, while one copy of *scs* ^*ae*^ is sufficient to re‐establish vital and plump seeds.

The most studied result of alloplasmy is cytoplasmic male sterility, a phenomenon exploited in plants to produce commercially superior hybrids (Cisar and Cooper, 2002). However, the effect of the *scs* genes is not to restore male fertility in hybrid combinations, but rather to re‐establish the compatible interaction between the nucleus and the alien cytoplasm. This is defined by the overall vigour of the progenies, rather than their fertility. The presence or absence of the *scs* locus can be determined phenotypically by observing the plump or shrivelled nature of alloplasmic seeds, respectively (Figure 1). Deleted *scs* ^*ae*^ (∆*scs* ^*ae*^) shrivelled seeds fail to germinate naturally and to date have not been germinated on media or with hormone treatment (Michalak *et al*., 2013). Michalak *et al*. (2013) pinpointed the location of the *scs* ^*ae*^ gene to a 12.9‐centi‐rays (cR, map unit) region on chromosome 1D of wheat by means of *in vivo* radiation hybrid (RH) mapping (Hossain *et al*., 2004a; Kalavacharla *et al*., 2006; Michalak *et al*., 2013). In wheat, viable RH lines can be produced, and the deletion caused on the genome by the radiation treatment can be exploited to investigate associations between markers and mutated phenotypes (reviewed in Kumar *et al*., 2014). Here is presented an expansion of that work, which allowed ‘landing’ to a single‐gene interval by means of ‘fast‐forward positional cloning’ (Schneeberger and Weigel, 2011; Trick *et al*., 2012).

## Results

### Bulk segregant analysis by sequencing of radiation hybrids (bulkSeq) to refine the *scs* ^*ae*^ interval

**Figure 2:**
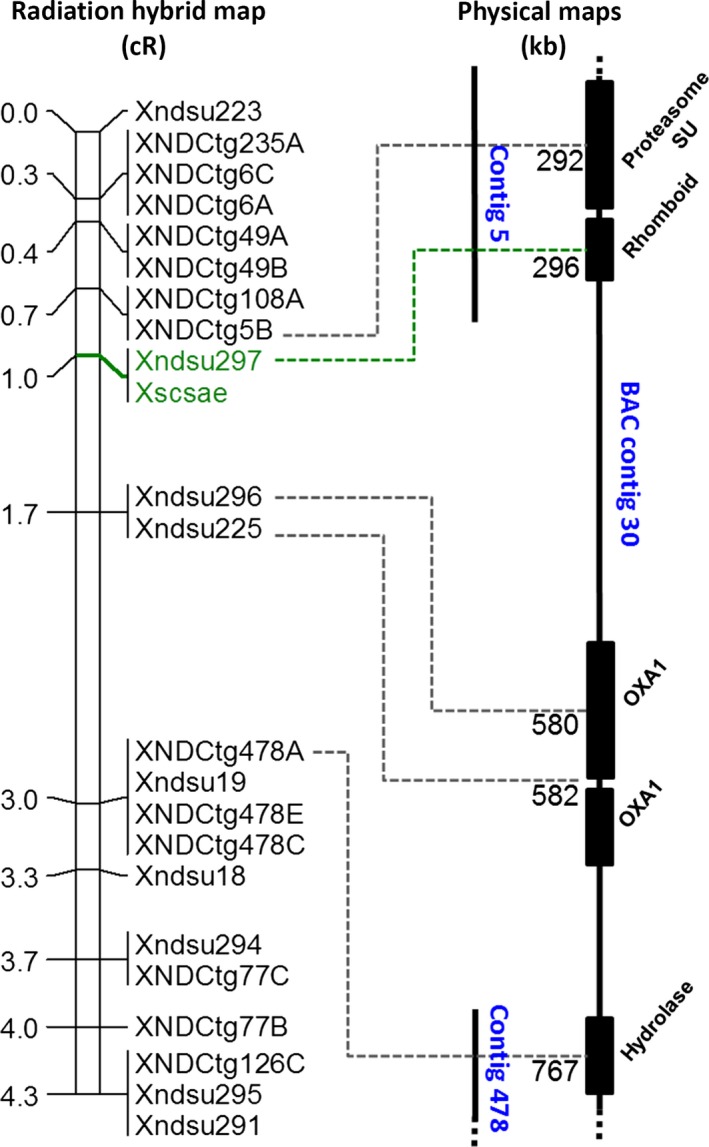
Fine‐mapping via radiation hybrid and the physical interval of the *scs* ^*ae*^ locus. The number to the left of the radiation hybrid map indicates the cR position of the markers. The marker cosegregating with the *scs* ^*ae*^ phenotype is depicted in green. The BAC contig 30 assembly is represented to the right as a vertical black line, and the length of the line is proportional to its physical size. The position of two contigs of extended reads derived from the bulkSeq analysis is reported in the middle and aligned to the continuous sequence of BAC contig 30. Genes are represented by solid black boxes, and their Pfam description is reported diagonally. The physical position amplified by the forward primer of each marker is indicated on the contigs as dashed lines, and the position in Kb on BAC contig 30 is reported.

In wheat, viable RH panels can be generated, phenotyped, genotyped and used in forward genetic studies. The DNA of one subset of six informative RH lines showing the ∆*scs* ^*ae*^ phenotype was mixed to create a negative bulk (bulk^NEG^), while the DNA of six RH lines showing the *scs* ^*ae*^ phenotype was mixed to create a bulk^POS^ (Table S1). These bulks were genotyped by sequencing, after a step of genome complexity reduction by cleavage with two restriction enzymes (*Pst*I and *Aat*II). Sequencing of the *Pst*I library for bulk^POS^ and bulk^NEG^ produced ~62‐m good‐quality pair‐end reads for a total of 12.4 Gb of sequences. The *Aat*II restriction library was mildly contaminated with an unidentified prokaryote; following the removal of these foreign reads ~48‐m good‐quality pair‐end reads for a total of 9.6 Gb of sequences were obtained. The short reads were assembled into ‘contigs of reads’. The average sequencing coverage was 49× (reads divided by contigs) for the *Pst*I library and 73× for the *Aat*II library. In total, 1 919 219 ‘contigs of reads’ were investigated for association with the *scs* ^*ae*^ phenotype, and 3318 were identified in the bulk^POS^, but were deleted in the bulk^NEG^. These are expected to span the interval of the *scs* ^*ae*^ locus as defined by the six RH lines that compose the bulk ^NEG^ (Table S1). In other words, they are present only in the RH lines carrying the *scs* ^*ae*^ locus (bulk^POS^) but are missing in the RH lines with the *scs* ^*ae*^ locus deleted (bulk^NEG^). The 3318 ‘contigs of reads’ were extended taking advantage of the 52× survey sequence of the *A. tauschii* genome (Luo *et al*., 2013; You *et al*., 2011). An iterative *in silico* approach was used to iteratively query the *A. tauschii* survey sequence with the short reads, identify matching sequences, assemble these and repeat with the extended sequences. The result was 3318 ‘contigs of reads’ extended from 100 to 300 bp to 1–10 Kbp. These longer sequences were assembled into 556 ‘contigs of extended reads’ with an average length of 2130 bp (Table 1). The resulting gapped assembly of the *scs* ^*ae*^ region had a N50 of 66, L50 of 2896 bp and spanned 1.15 Mb (Table 1). The largest contig spanned ~70 Kb, while the shortest just 138 bp. The 556 contigs were annotated to find 45 open reading frames (ORFs) corresponding to genes previously identified in at least one of the model species, for an average frequency of one gene every 25 Kb (Table S2 and Table S3). Twenty‐four genes showed colinearity with the expected regions of rice (*Oryza sativa* L.) chromosome 10, *Brachyopdium distachyon* Eauv. chromosome 3 and sorghum (*Sorghum bicholor* Moenc.) chromosome 1 (Luo *et al*., 2009; Michalak *et al*., 2013) (Table S2). The remaining 21 confirmed ORFs correspond to genes outside the synthenic interval (Table S3). From the sequences of these contigs containing genes were developed 22 markers, which were then used to genotype a RH population of 644 lines. The resulting map spans 4.3 cR distributed in 11 unique loci. This resolution was sufficient to orient the eight ‘contigs of extended reads’ that contained ORFs in a continuous gapped scaffold (Figure 2).

**Table 1:** Summary of the assembly of the extended Illumina bulk^POS^‐specific contigs

Data (unit)	Value
L50 (bp)	2896.0
N50 (#)	66.0
Total length (bp)	1 148 578.0
Average length (bp)	2130.0
Max length (bp)	70 049.0
Min length (bp)	138.0
Average coverage (X)	2.3
Contigs (#)	556.0
ORF (#)	916.0
Confirmed ORF (#)	45.0
ORF syntenic	24.0
Contigs with syntenic ORF (#)	8.0
ORF nonsyntenic (#)	21.0
Contigs with nonsyntenic ORF (#)	18.0

#, number.

The RH map allowed to further refine the position of the *scs* ^*ae*^ locus to the 1 cR interval between markers *XNDCtg5B* and *Xndsu296* (Figure 2). A single marker (*Xndsu297*), located 0.3 cR distal of X*NDCtg5B* and 0.7 cR proximal of *Xndsu296,* was found to cosegregate with the phenotype in all 644 tested lines (Figure 2). This interval was represented by the ‘contigs of extended reads’ number 5 and 478.

### Physical mapping of the *scs* ^*ae*^ interval

The survey sequence of the physical map of *A. tauschii* was searched for Bacterial Artificial Chromosome (BAC) contigs that span this 1 cR interval using the ‘contigs of extended reads’ number 5 and 478 as query. A single contig (ID number: 30) was identified as containing the *scs* ^*ae*^ locus. Sequencing by minimum tiling path and assembly by means of nano‐mapping of this contig resulted in a continuous sequence of 1.83 Mb containing 15 predicted ORFs, or one ORF every 122 Kb. The ‘contigs of extended reads’ 5 and 478 aligned perfectly to the assembled sequence of this BAC contig. Marker *XNDCtg5B* tags position 292 Kb of BAC contig 30, *Xndsu297* tags position 296 Kb, and *Xndsu296* position 580 Kb (Figure 2). Markers *XNDCtg5B* and *Xndsu296* are separated by 1 cR, which indicates a map resolution of 288 Kb. Furthermore, only one ORF was predicted within the interval pinned by the flanking markers, and this ORF is tagged by *Xndsu297*, the only marker co‐segregating with the *scs* ^*ae*^ phenotype. This ORF is a putative *rhomboid* gene, surrounded by a *hypothetical proteasome subunit* gene 490‐bp proximal of its starting codon, and on its distal end by 280 Kb of noncoding sequence that terminates into a series of multiple putative *OXA1* genes. Given that *XNDCtg5B* excludes the *proteasome subunit* ORF from the *scs* ^*ae*^ locus, and that *Xndsu296* excludes the *OXA1* ORFs, this positional cloning study resulted in ‘gene‐landing’, leaving the *rhomboid* ORF as the only candidate for the *scs* ^*ae*^ locus based on the *A. tauschii* genome. Further, the ‘contigs of extended reads’ 5 and 478 are derived from direct sequencing of chromosome 1D of bread wheat and also do not indicate the presence of any other ORF within this marker interval other than the *rhomboid*. This confirmed that this region of the genome does not appear to differ between bread wheat and *A. tauschii*.

### A nonsynonymous homoeologous sequence variant (HSV) among the homoeoalleles of the *rhomboid* gene

The genomic region corresponding to this *rhomboid* was resequenced from 207 bp upstream of its start codon to 367 bp downstream of its stop codon, for a total length of 1944 nucleotides. Due to its vital role, the *scs* gene is conserved among varieties; therefore, the sequencing was rather performed on different genomes and species to identify meaningful variation. The sequence was obtained from the DNA of *T. aestivum* Chinese Spring flow‐sorted chromosomes 1A, 1B and 1D, as well as from a different accession of *A. tauschii*, two alloplasmic lines with *A. longissima* (lo) cytoplasm that carry a functional homoeologous of *scs* ^*ae*^ derived from *T. timopheevi* on their chromosome 1A: (lo)*scs* ^*ti –*^ and (lo)*scs* ^*ti*^ *scs* ^*d*^, *T. durum* (1A + 1B) and *T. durum* + 1D [*T. durum* var ‘Langdon’ 1D(1A)]. This represents a complete set of genotypes carrying various versions of *scs*.

**Figure 3:**
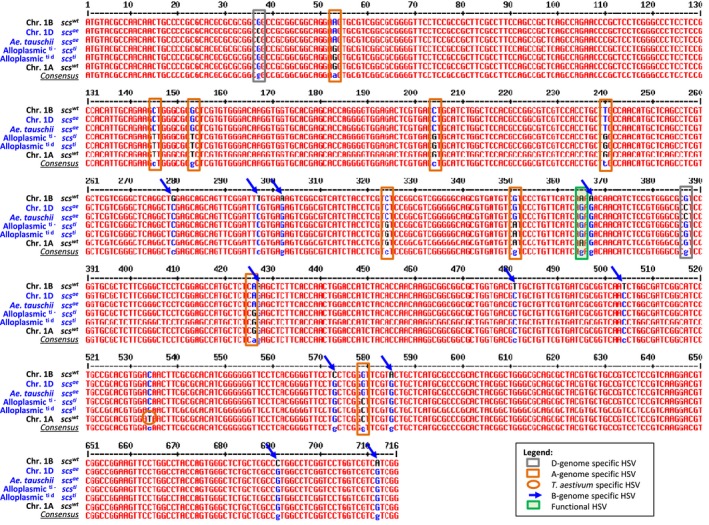
Haplotype differentiation between the expressed portion of homoeologous versions of the *rhomboid* gene tagged by *Xndsu297*. The *scs*‐bearing genotypes are presented in blue colour, and the allele at their *scs* locus is indicated; the ∆*scs* genotypes are shown in black. For clarity, both *scs* ^*ae*^ and *scs* ^*ti*^ can restore compatibility to the (lo) cytoplasm and were shown to be homologous. The legend provides indication on how to decipher the various homoeologous sequence variant. Chr., chromosome; Alloplasmic, *Ae. longissima* cytoplasm.

**Figure 4:**
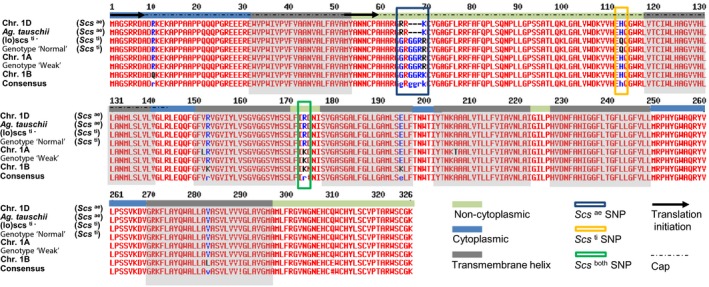
Alignment of the amino acid sequences translated from the *rhomboid* gene tagged by *Xndsu297*. The black arrows indicate the two possible origins of translation. The regions corresponding to transmembrane helices are shaded in grey, and the intertransmembrane portions are colour‐coded to indicate whether they surface in the cytoplasm (light blue) or to the internal side of the membrane (light green). The homoeologous sequence variations that produce changes in the amino acid sequence are boxed and colour‐coded. The cap region is indicated with a dashed black line.

Gene annotation revealed five exons and four introns. The sequences obtained by Sanger sequencing matched precisely the ‘contig of extended reads’ 5 and BAC contig 30 assemblies. The alignment of the sequences obtained for the *rhomboid* gene from various homoeologous revealed several homoeologous sequence variants (HSVs) in the coding sequence (Figure 3). The D‐genome of *A. tauschii* and *T. aestivum* was discriminated from the A and B by 2 HSVs, the B‐genome by 11, the A‐genome by 9 and one HSV discriminated the *T. aestivum* A‐genome from the *T. durum* A‐genome. More importantly, one single HSV discriminated genotypes harbouring the functional *scs* loci (*scs* ^*ae*^ or *scs* ^*ti*^) from those with the nonfunctional or wild‐type *scs* gene (∆*scs* or *scs* ^*wt*^). The HSV carried by the *scs*‐bearing lines was a guanine (G) at position 997 of the gDNA, while the ∆*scs* haplotypes had an adenine (A) (Figure 3). As the HSV discriminated between the locus on different homoeologous chromosomes, its two states (‘G’ or ‘A’) were considered as homoeoalleles. The coding sequence of the *rhomboid* gene generates a protein with two possible start codons in frame that produce a 326 or a 274 amino acid protein (Figure 4). The identified ‘G’ to ‘A’ HSV results in a nonsynonymous change of the 173rd amino acid from an arginine (ARG or R) to a lysine (LYS or K) (Figure S5). This amino acid sequence corresponds to a membrane protein with seven transmembrane domains and is recognized by InterProScan as a *S54 rhomboid peptidase* (ID: IPR002610).

### Expression analysis of the *rhomboid's* homoeoalleles

**Figure 5:**
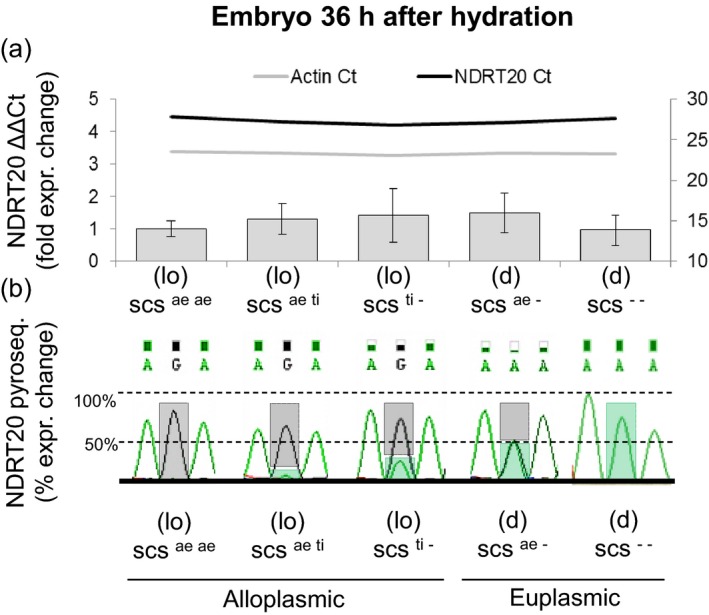
Expression of the *rhomboid* gene tagged by *Xndsu297* in embryonic tissue 36 h after hydration under alloplasmic and euplasmic conditions. (a) Relative quantification of *Xndsu297 by* NDRT20 by comparison with an actin gene. The samples are labelled with their alloplasmic (lo) or euplasmic (d) definitions, as well as their dosages of the *scs* genes, with ‘‐’ indicating a null allele. The expression changes are provided as fold differences of the average of the replicates, and the error bars represent their standard deviations. The threshold cycles (Ct) are provided for comparison as colour‐coded horizontal lines with their values reported on the secondary Y axis to the right. NDRT20 amplifies all of the *rhomboid* homoeoalleles. (b) Pyrosequencing discrimination of the homoeoalleles amplified by NDRT20. The height of the sequencing peaks is reported in their raw format as calculated by ChromasPro software. The height of the bars represents the relative abundance in the cDNA amplicon of the ‘G’ nucleotide (grey) vs. the ‘A’ nucleotide (green) at the nonsynonym homoeologous sequence variant position. Refer to the Material & Methods section for a more detailed description of the codes used for the wheat lines and their *scs* homoeoalleles.

Two primers (NDRT20) were designed on separate exons to amplify across the HSV. Two time frames were selected for expression analysis: (i) leaves of 15‐day‐old seedlings, a stage of growth that cannot be reached by ∆*scs* genotypes with alien plasmons; and (ii) embryos dissected from seeds exposed to 36 h of hydration, this is a critical step for germination and mRNA could be collected from both *scs* and *∆scs* genotypes. Real‐time quantitative reverse‐transcription PCR (qRT‐PCR) revealed no significant difference in expression of the gene in embryo or leaf tissue between alloplasmic and euplasmic lines with varying dosages of the *scs* genes (Figure 5; Figure S1). However, pyrosequencing of six amplicons from each sample used for qRT‐PCR revealed a clear change in the type of *rhomboid* homoeoalleles that were expressed (Figure 5). The six replicates generated nearly identical chromatographs, with normalized G/A peaks of the same height. Four expression classes could be clearly distinguished on the basis of changes in ‘G’‐type and ‘A’‐type amplicons abundance: (i) the presence of two copies of *scs* in alloplasmic conditions maximized the expression of the ‘G’ form; (ii) the hemizygous (one copy) condition of *scs* in the alloplasmic state allowed low expression of the ‘A’ form, which was largely overpowered (>3 : 1) by the ‘G’ type; (iii) the same hemizygous state of *scs*, but in euplasmic conditions, permitted both versions to be equally expressed; and (iv) in the absence of the ‘G’ version in the euplasmic condition, only the ‘A’ form was expressed. As mentioned, it was not possible to test the expression of the ‘A’ version in alloplasmic condition in the leaf tissue due to lethality of lines without *scs*. Unfortunately*,* in shrivelled seeds [(*lo*) *∆scs* genotypes], the mRNA degradation is too high even just 36 h after hydration that it was not possible to ensure proper qPCR amplification.

### Phyletic evolution of the rhomboid across the plant kingdom

**Figure 6:**
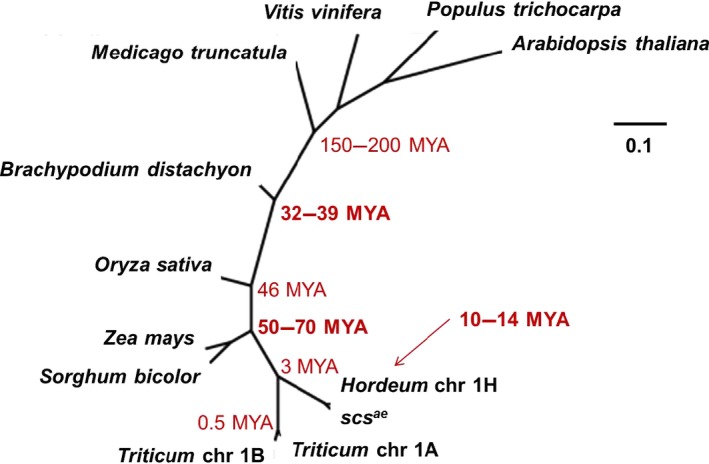
Phylogenetic comparison of rhomboid Expressed Sequence Tags (ESTs) from across the plant kingdom confirms its phyletic evolution. The value for evolutionary distance in millions of year was derived from Paterson *et al*. (2009).

The plant protein databases available on NCBI were searched using the aminoacid sequence of the protein predicted for the 1D version of the rhomboid. BlastP and BlastX searches of *Hordeum vulgaris*,* Brachypodium distachyon*,* Vitis vinifera*,* Arabidopsis thaliana*,* Zea mays*,* Sorghum bicolor*,* Oryza sativa*,* Medicago truncatula* and *Populus trichocarpa* identified good matches. ClustalW was used to estimate the distance between the proteins (list and E‐values available in Table S6) and visualized using Phylodendrum 0.8d (http://iubio.bio.indiana.edu/webapps/Phylodendron/). The result is displayed in Figure 6, with superimposed evolutionary distances as estimated by Paterson *et al*. (2009). The tree shows a near complete agreement with the known evolution of the grasses, with the exclusion of *Zea mays* and *Sorghum bicolor*, which have hypothetically diverged from *Triticum* before *Oryza* and *Brachypodium*. Nevertheless, it is clear the phyletic evolution of this rhomboid gene across the plant kingdom, with the length of branches that is shorter than expected when compared to the actual speciation distances, as branches’ lengths are near identical between all species. Furthermore, it is interesting to notice how the *Hordeum vulgaris* chromosome 1H version of rhomboid highly resembles the *scs* ^*ae*^ gene of chromosome 1D.

## Discussion

### Fast‐forward genetics for ‘gene‐landing’ on the *scs* locus

The *in vivo* RH strategy has been documented in wheat to provide high map resolution (reviewed in Kumar *et al*., 2014, 2012a, b; Bassi *et al*., 2013). Here, the resolution achieved was 288 Kb, which is fivefold higher than what previously reported by Michalak *et al*., 2013 (1.5 Mb) using a population 3.5 times smaller. Further, this resolution is similar to what reported by Bassi *et al*. (2013), where a resolution of 388 Kb was achieved for chromosome 3B using a population of 696 RH individuals tagged with 140 markers. Overall, this is the highest map resolution reported to date for RH in wheat, confirming the advantages of this approach for mapping its complex genome.

RH BulkSeq generated 60 million reads from two libraries, which queried the large genome of durum wheat at ~2 million sites and assured the rapid identification of 21 ORFs within the interval. Trick *et al*. (2012) reported the use of BulkSeq targeting the cloned *grain protein content B1* (GCP‐B1) gene in durum wheat by means of RNA sequencing of bulks of isogenic lines. Their scan of 25 K genes with 40 million reads revealed 64 ORFs that were differentially expressed between the two bulks. Using Single Nucleotide Polymorphisms (SNPs) derived from these putative genes for genotyping a recombinant population pinpointed the locus to an interval of 0.4 cM. In a similar experiment targeting Yr15, Ramirez‐Gonzalez *et al*. (2014) using BulkSeq by RNASeq reported that approximately 40 million reads were used to query 27 K genes and obtain 35 ORFs spanning the interval. Further fine‐mapping reduced the locus interval to 0.77 cM, which contained two SNPs useful for breeding purposes. In comparison, RH BulkSeq generated a larger number of good‐quality reads from the same number of HiSeq lanes. This is probably due to the higher thermo‐stability of DNA, as compared to the RNA step necessary for RNASeq. Further, the use of genome complexity reduction allowed the investigation of nearly 2000 K loci, as compared to 25 and 27 K genes queried by RNA BulkSeq. Moreover, the use of DNA instead of RNA does not require *a priori* knowledge of the expression pattern of the targeted gene. In Trick *et al*. (2012), the authors indicated that the functional SNP for GPC‐B1 could not be identified because it was not expressed in the leaf tissue sampled for the experiment. Nevertheless, the resolution achieved by the three approaches was comparable with 64 ORFs identified by Trick *et al*. (2012), 35 ORFs by Ramirez‐Gonzalez *et al*. (2014) and 22 ORFs by us. However, the one reported here is the only study in wheat that granted ‘landing’ to a single candidate gene. This is likely due to the higher resolution provided by RH map as compared to recombination‐based approaches. In fact, all the contigs bearing genes (Alnemer *et al*., 2013; Goff *et al*., 2011) were ordered into a scaffold spanning the genomic region of *scs* ^*ae*^ by means of RH. Furthermore, the resolution was sufficient to pinpoint the *scs* ^*ae*^ locus to a region of just 288 Kb containing only one ORF: a putative *rhomboid* gene. Therefore, it appears strategic the combination of the map resolution provided by RH with the detailed genotyping scan by BulkSeq. However, RH‐mediated genetic dissection may not be suitable for all types of phenotyping. In fact, the hemizygous nature of RH lines may cause weakness under field conditions when certain genes are deleted and preclude genetic dissection of agronomic traits (reviewed in Kumar *et al*., 2014). In those cases, approaches such as that presented by Ramirez‐Gonzalez *et al*. (2014) by BulkSeq of mRNA pools would be more suitable. Especially interesting are also the cases of using DNA BulkSeq of contrasting recombinant inbred lines, as achieved for rice (Takagi *et al*., 2013) or chickpea (Das *et al*., 2015) and defined as ‘QTL‐seq’. In the latter case, the map resolution provided by bulks of recombinants was sufficient to narrow the genomic interval to a region containing just six ORFs. While it remains to be confirmed the actual resolution that can be achieved by QTL‐seq in large polyploid genomes such as wheat, it is still evident that RH BulkSeq as shown here outperformed both methods in terms of resolution capacity. Kumar *et al*. (2014) summarized the advantage of employing RH over recombination‐based mapping techniques. Here, we would like to further state that the use of RH‐BulkSeq should be preferred over mRNA BulkSeq or QTL‐seq whenever phenotyping of RH lines is possible. When not possible, these other methodologies represent excellent strategies for Fast Forward Genomics.

### Functional characterization of the *rhomboid* gene

The *rhomboid* gene identified as the *scs* ^*ae*^ locus contains five exons and four introns. A single HSV (‘G’ to ‘A’ nucleotide change) within the coding sequence differentiates the *scs* ^*ae*^ and the *scs* ^*ti*^ haplotypes from the ∆*scs* types (Figure 4) resulting in a nonsynonymous mutation (R→K) in the amino acid sequence. This *rhomboid* gene showed constitutive expression in both the embryos during seed germination (Figure 5) and 15‐day‐old seedlings (Figure S1). Interestingly, the mRNA expression of the *rhomboid* was only slightly lower than the housekeeping gene *actin,* and its constitutive expression is consistent with previous microarray studies of various tissues in wheat (Schreiber *et al*., 2009). Pyrosequencing of the cDNAs revealed that the two *rhomboid* homoeoalleles (‘G’ and ‘A’ types) are differentially expressed depending on the alloplasmic or euplasmic status of the seeds (Figure 4), with a preference for the ‘G’‐type when the *T. durum* nucleus is immersed in the (lo) cytoplasm. As the *scs* genes control nuclear–cytoplasmic compatibility within all cells, at all life stages of an organism, these loci are expected to maintain their activity throughout the life of the plant in all tissues. Finally, differential expression is expected between the various *scs* homoeoalleles depending on the cytoplasmic genome and the NCI.

A nonsynonym HSV was identified in the coding sequence of this *rhomboid* gene, which translates into an arginine in the protein derived from the *scs* ^*ae*^ or the *scs* ^*ti*^ homoeoallele (‘G’ or ARG types), to a lysine in the non‐*scs* homoeoallele (‘A’ or LYS type). Both of these amino acids are basic, with a positive charge that is distributed throughout the guanidino group in ARG and concentrated on the amino group in LYS. Basic‐to‐basic amino acid mutations do not appear to cause major changes in the overall protein structure, and ARG to LYS are among the most common substitutions in nature. However, an ARG to LYS substitution has been proved multiple times to be the key factor causing drastic functional modifications in different types of proteins (Betts and Russell, 2003; Klumpp *et al*., 2003; Mills *et al*., 2005; Ryan and O'Fagain, 2007). Rhomboids influence many cellular regulations, and their function has been postulated to occur through signal emission by the release of transcription factors from the organelles membranes (for reviews, see Urban, 2006; Freeman, 2008; and Knopf and Adam, 2012). From a plant prospective, it is worth mentioning that at least 10% of the transcription factors of *Arabidopsis thaliana* are membrane bound and require a mechanism similar to that described for the rhomboid to be released and reach their targets (Seo *et al*., 2008). It remains to be determined how this candidate rhomboid gene could control the compatible or incompatible NCI on the basis of this single amino acid change, but the outcome of this study is an ideal starting point to investigate further this complex protein family.

### The ‘Maan's scs hypothesis’ supports the *rhomboid* as candidate gene

The first scientist to discuss the biological importance of *scs* genes in controlling NCI stated, in what has become known as the ‘Maan's *scs* hypothesis’ (Maan, 1975; Maan and Endo, 1991; reviewed in Michalak *et al*., 2013), that (i) the origin of the *scs* genes pre‐dates the speciation of the grasses, (ii) that allelic copies are present in all grass diploids to maintain vital NCI and (iii) that once interspecific hybridization occurs between diploids only the *scs* version carried by the maternal progenitor (cytoplasm donor) provides NCI, while the paternal version becomes free to accumulate mutations. It is then compulsory to review the available information regarding the *rhomboid* in the prospective of ‘Maan's *scs* hypothesis’.

It has been suggested that the rhomboid gene family is derived by phyletic evolution from an ancestral form that dates back to the last common eukaryotic progenitor (Lemberg and Freeman, 2007). In this sense, (i) the origin of this *rhomboid* gene would pre‐date the speciation of the *Poidea* family as suggested by Maan. Thus, it is likely that the rhomboid gene identified here functions similarly in regulating NCI and speciation of various wheat species. Furthermore, a similarity study of rhomboid Expressed Sequence Tags (ESTs) from across the plant kingdom well supports its phyletic evolution (Figure 6), and therefore, raises the questions of whether the rhomboid controls NCI in species other than wheat as argued by Lemberg and Freeman (2007) and Maan himself. Urban (2006) in a comprehensive review of the rhomboids noticed how their regulatory functions were conserved between metazoan and the animal kingdom, a very rare evolutionary event. With the data presented here, we would expand the question expressed by Urban (2006) to also comprehend the plant kingdom.

Finally, the expression characterization revealed that an active copy of this *rhomboid* exists on all homoeologous chromosomes of wheat, and depending on the specific cytoplasm each copy is preferentially expressed. If the hypothesis formulated by Maan is correct, (ii) then the 1B version of the *rhomboid* gene should be of maternal origin and the most important allele to maintain NCI in the B plasmon of wheat (tetraploid and hexaploid). Also, the 1D version provides proper NCI in combination with the *A. longissima* plasmon ‘S’, indicating that *A. tauschii* (plasmon ‘D’) and *A. longissima* share a common maternal ancestor from which *scs* ^*ae*^ originated. The data presented here support the *rhomboid* gene involvement in directing NCI in alloplasmic wheat as the *scs* gene.

## Conclusion

The *scs* ^*ae*^ locus operated a critical function in the evolution of grass species. Here we have reported a successful attempt in refining its genomic location by deploying RH and sequencing of contrasting bulks. To the best of our knowledge, this is the first time that these methodologies have been combined. The exploitation of additional novel technologies such as nano‐mapping and *ad hoc* algorithms, together with the availability of unique alloplasmic germplasm, has made possible achieving ‘gene‐landing’ to a single candidate. Further, a single nucleotide mutation was identified as the possible variation controlling the correct communication between the nucleus and the cytoplasm of several grass species. Attempts at confirming this outcome by means of transformation are currently underway, but these will require overcoming specific challenges considering the alloplasmic and therefore recalcitrant nature of the germplasm studied here. Still, the bundle of methodologies presented should be readily used in other map‐based cloning, especially when targeting traits that lack any available genetic diversity, as it was the case for *scs* ^*ae*^.

## Material and methods

### Plant material and phenotyping

The plant material, population development and phenotyping methods have been previously described in detail by Michalak *et al*., 2013. Briefly, a radiation hybrid (RH) population of 644 individuals was developed through the crossing of *T. durum* var. ‘Langdon’ (LDN) and its aneuploid 1D line LDN 1D(1A) irradiated at 150 Grey (Gy). The RH population was screened at seedling stage with markers ndsu212, ndsu21 and ndsu3 (described in Michalak *et al*., 2013) searching for lines that presented a breakage between these loci (a.k.a. ‘recombination events’ in genetic mapping studies). The selected RH_1_ lines were then transplanted into soil, grown under greenhouse controlled conditions and used as pollen donors for crossing with the (*lo*)*scs* ^*ti*^ *–* line, an alloplasmic tester line with *A. longissima* cytoplasm and hemizygous for *scs* ^*ti*^, the homoeoallele of *scs* ^*ae*^ from *T. timopheevii*. The ratio of shrivelled to plump seeds obtained by test‐crossing was converted into the deletion‐type (a.k.a. ‘haplotype’ in genetic mapping studies) for the *scs* ^*ae*^ locus, where a 1 : 1 ratio indicates that the *scs* ^*ae*^ gene has been deleted, while 1 : 3 indicates retention of the locus. Additionally, seeds of *A. tauschii* were kindly provided by Dr. Steven Xu (USDA‐ARS). Further, LDN and its aneuploid derivative LDN 1D (1A) (Joppa and Williams, 1988) also were employed as euplasmic lines, referred to as (d)*scs* ^*−*^ *–* and (d)*scs* ^*ae ae*^, respectively. The alloplasmic lines of durum employed here had the cytoplasm of *T. durum* replaced by the cytoplasm of *A. longissima* (*lo*). Four alloplasmic genotypes have been used, each with a different combination of *scs* homoeoalleles, two in hemizygous condition: (lo)*scs* ^*ti*^ – and (lo)*scs* ^*ae*^ ‐; one in heterozygous condition (Gehlhar *et al*., 2005): (lo)*scs* ^*ti d*^, and a double hemizygous [(lo)*scs* ^*ae*^ *– scs* ^*ti*^ *–* or (lo)*scs* ^*ae ti*^ for simplicity] obtained by crossing (lo)*scs* ^*ti*^ *−* to (d)*scs* ^*ae ae*^ followed by marker confirmation of the presence of 1A^ti^ and 1D. For simplicity, the lines are described in the text using only their cytoplasmic and *scs* designations.

### Genotyping by PCR

The North Dakota State University (ndsu) marker amplification conditions and primer sequences have been previously presented by Michalak *et al*., 2013. These are STS markers developed based on EST sequences of wheat genes orthologous to rice, *Brachypodium* and sorghum. The specificity for the 1D version of the marker is supported by a difference in size of the amplified fragment, detected using high‐resolution polyacrylamide gel, or by primers designed on HSV identified through sequencing of flow‐sorted chromosomes (Dolezel *et al*., 2009).The North Dakota Contig (NDCtg) markers were obtained by designing primers (see Table S4 for sequences) on noncoding portions of the assembled contigs. All primers were amplified at the same conditions described in Michalak *et al*., 2013 but using 2.5 mM of MgCl_2_ and 65–60 °C touch‐down annealing temperature. The chromosome 1D‐specific polymorphism was detected on 2% agarose gel, typically represented by the lack of a fragment in genotypes that do not carrying chromosome 1D (i.e. LDN) and a distinct fragment in 1D genotypes (i.e. LDN 1D(1A)). Sequences of the primers proving 1D‐specificity are reported in Table S4. All the 1D‐specific markers were then used in combination (i.e. multiplex PCR) with the control marker DEASY (Kumar *et al*., 2012a) to genotype the most informative RH‐1D lines. A map was generated using the Carthagene software (de Givry *et al*., 2005).

### Sequencing, assembly and annotation

A complete description of the procedures followed for sequencing, assembly and annotation is provided as supplementary information.

### Resequencing of the rhomboid gene tagged by *Xndsu297* and HSV detection

The TC sequence associated with marker *Xndsu297* (Ctg5) was orthologous to a *rhomboid* gene in the three sequenced model species. Six primer pair combinations (defined as RHBD1‐6) were designed on the basis of the sequence of the contig that carries the marker (Ctg5) to span from 207 bp upstream of the gene to 367 bp downstream. The sequences of these primers are made available in Table S4, and their amplification conditions are as reported for the NDCtg markers. The primers were used to amplify the DNA of the flow‐sorted chromosomes 1A, 1B and 1D (Dolezel *et al*., 2009), and the band corresponding to the expected amplicon size was eluted from the gel and sent for sequencing at GeneWiz (South Plainfield, NJ). The obtained sequences were first displayed and analysed with ChromasPro (Technelysium Pty Ltd, Helensvale, Qld, Australia) to assure good quality, and then assembled into a continuous sequence for each chromosome. The same sequence was obtained by amplification of *A. tauschii*, (lo)*scs* ^*ti−*^, (lo)*scs* ^*ti d*^, *T. durum* [(d)*scs* ^*− −*^] and LDN 1D(1A) [(d)*scs* ^*ae ae*^]. These genotypes are, with the exclusion of *A. tauschii*, tetraploid, and direct sequencing would fail. So the amplicons obtained were subcloned into pGEM‐T Easy vector (Promega, Madison, WI) following the provider guidelines, and then sequenced. On the basis of sequence similarity with the flow‐sorted chromosomes 1A, 1B and 1D sequences, the clones were discriminated for their homoeologous origin. For *T. durum,* the 1A version was used for downstream comparisons, while for all other genotypes, the 1D type was considered more informative. All sequences were aligned using the ‘MultAlin’ online tool (available at http://multalin.toulouse.inra.fr/multalin/; Corpet, 1988), and significant HSVs were searched visually.

### Expression analysis

Plump seeds of (lo)*scs* ^*ae ae*^, (lo)*scs* ^*ae ti*^, (lo)*scs* ^*ti*^ *–*,* (d) scs* ^*ae*^ *–* and *(d) scs* ^*−*^ *–* were hydrated for 24 h at 4 °C in the dark, then transferred at room temperature to initiate germination. After 12 h, the embryos were dissected with a scalpel and collected into liquid nitrogen. The embryos were ground using a mortar and pestle, making sure to avoid thawing of the tissues. Equal weights of ground tissue were used for mRNA extraction by Qiagene mRNA Extraction Kit following the provider guidelines. Also, the same genotypes were allowed to germinate, and 15 days after initial hydration the tip of the first leaf was collected in liquid nitrogen for mRNA extraction. The M‐MLV Reverse Transcriptase Kit (Promega) with Oligo dT and rRNAse inhibitor was used to generate cDNA, following the provider instructions. The primers NDRT20 FW and NDRT20 RW (sequence available in Table S4) were designed to amplify a 197‐nucleotide cDNA fragment comprised between two exons, corresponding to 284 bp in the gDNA (87‐bp intron). This fragment contains a nonsynonym HSV which discriminates between *scs* and ∆*scs* genotypes. As a reference, a primer combination was designed to amplify 124 bp of an actin gene (GeneBank ID: TA411_4571). The Roche 2× SybrGreen Fast Start Mastermix was used to amplify the cDNA samples in a 20‐μL reaction with 0.5 μm of NDRT20 or actin primers and 400 ng of cDNA. The samples for the qPCR were run in triplicate on a 7500 Fast Real‐Time PCR System (Applied Biosystems, Carlsbad, CA) at 50 °C for 2 min, 95 °C for 10 min, followed by 40 cycles of 95 °C for 15 s, 60 °C for 1 min. The data were analysed with the SDS v2.2.1 software (Applied Biosystems, Carlsbad, CA) employing the 2^−ΔΔCt^ equation (Livak and Schmittgen, 2001) to estimate the relative quantification (RQ) of the NDRT20 expression. The threshold cycle was automatically determined by the SDS software, and the (lo)*scs* ^*ae ae*^ sample was set as the normalizer. An attempt was made to design two TaqMan probes (available in Table S4) targeting each a specific version of this HSV. Unfortunately, these two probes in combination with the TaqMan Fast Advanced Master Mix (Applied Biosystems) and primers NDRT20 failed to provide adequate emission profiles, and were then discarded. Instead, the relative level of expression of the two homoeoalleles was determined by direct pyrosequencing. New amplicons were generated by qPCR by stopping the reaction in its exponential phase at cycle 28. The products were run on agarose gel, eluted and sent for pyrosequencing at GeneWiz. The chromatograms were analysed using ChromasPro. The heights of the two adenine peaks surrounding the HSV position were measured and then averaged. The heights of the guanine and adenine peaks at the HSV position were also measured. The height of the HSV peak divided by the average height of the surrounding peaks was considered as the normalized value. A value of 100% was given to the height of the guanine peak in (lo)*scs* ^*ae ae*^, and the relative expression of the other samples was calculated as compared to its value. To verify the quality of the analysis, all samples were sequenced three times from two biological replicates, and identical results were obtained for all replicates.

### Protein prediction

All the genomic sequences from homoeologous chromosomes 1A, 1B and 1D were used as input for GeneScan (available at http://genes.mit.edu/GENSCAN.html; Burge and Karlin, 1997) and the TriAnnot Pipeline (available at http://urgi.versailles.inra.fr/Tools/TriAnnot-pipeline; Leroy *et al*., 2011) employing the default parameters. The Coding DNA Sequence (CDS) and protein sequences predicted by these tools were aligned using ‘MultAlin’. The amino acid changes were visually identified in the alignment (Figure 5).The protein sequence from 1A, 1B, 1D and the other genotypes were searched by InterProScan (available at http://ebi.ac.uk/Tools/pfa/iprscan/; Quevillon *et al*., 2005) for similarities with previously annotate protein domains. Also, the SOSUI (available at http://bp.nuap.nagoya-u.ac.jp/sosui/; Mitaku *et al*., 2002) and TMpred (http://www.ch.embnet.org/software/TMPRED_form.html; Hofmann and Stoffel, 1993) tools were used to predict possible transmembrane helices (Table S5), while Phobius (http://phobius.binf.ku.dk/; Käll *et al*., 2007) was used to predict the protein cytoplasmic/noncytoplasmic orientation.

## Authors Contribution

FMB designed the research, performed the research, contributed new analytic, computational, etc., tools; analysed the data and wrote the paper. FG designed the research, contributed new tools and wrote the paper. YW, KL, SK, RD and MKMdJ performed the research and analysed the data. MJH and YQG performed the research, contributed new tools, analysed the data and wrote the paper. SWM and MM analysed the data and wrote the paper. SFK funded the research, designed the research, contributed genetic material, managed the project, analysed the data and wrote the paper.

## Conflict of interest

The authors declare no conflict of interest.

## Supporting information


**Figure S1** Expression analysis of the rhomboid gene by means of NDRT20 qPCR. The data are presented as relative quantifications comparison to an Actin gene. The samples are labelled with their alloplasmic (lo) or euplasmic (d) definitions, as well as their dosages of *scs* genes. The expression changes are provided as fold differences of the average of three replicates, and the error bars present their standard deviations. The threshold cycles (Ct) are provided for comparison as colour‐coded horizontal lines with their values reported on the secondary Y axis to the right.

**Table S1** Deletiontyping of the radiation hybrid (RH) lines used for bulk segregant analysis, modified from Michalak *et al*. (2013).
**Table S2** Contigs (Ctg) used for RH mapping, their gene content and orthologous relationships.
**Table S3** Contigs with pseudogenes.
**Table S4** Sequences of primers, probes, and adapters used in this study.
**Table S5** PDB Sum prediction of turning amino acids for transmembranes of the rhomboid protein (chr. 1D‐type). The amino acid (R) caused by the non‐synonymous HSV is indicated in bold.
**Table S6** NCBI protein entries identified across the plant kingdom as similar to *scs* ^*ae*^ and their reported alignment value to *scs* ^*ae*^ (*Triticum* chr 1D).
