# Supplementary figures and images for: Fast‐forward genetics by radiation hybrids to saturate the locus regulating nuclear–cytoplasmic compatibility in Triticum
Source: Plant Biotechnol J. 2016 Feb 24;14(8):1716–26. doi: 10.1111/pbi.12532 (PMC5067624; doi:10.1111/pbi.12532)

## Leaves 15 days after hydration

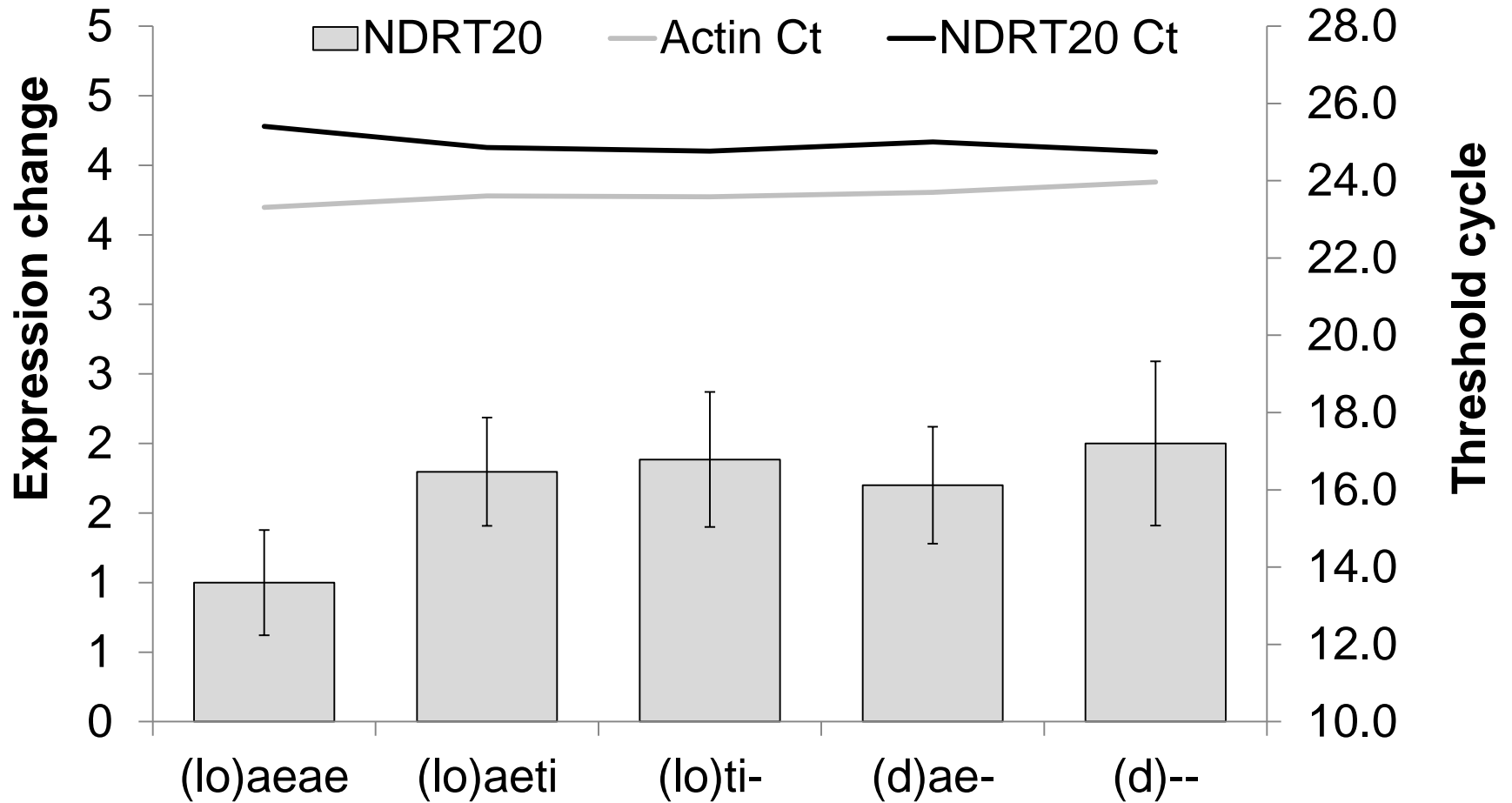

Supplement: Supplementary file 1 — Figure S1 Expression analysis of the rhomboid gene by means of NDRT20 qPCR. The data are presented as relative quantifications comparison to an Actin gene. The samples are labelled with their alloplasmic (lo) or euplasmic (d) definitions, as well as their dosages of scs genes. The expression changes are provided as fold differences of the average of three replicates, and the error bars present their standard deviations. The threshold cycles (Ct) are provided for comparison as colour‐coded horizontal lines with their values reported on the secondary Y axis to the right. [file PBI-14-1716-s001.pdf]
