# Supplementary material for: Fast‐forward genetics by radiation hybrids to saturate the locus regulating nuclear–cytoplasmic compatibility in Triticum
Source: Plant Biotechnol J. 2016 Feb 24;14(8):1716–26. doi: 10.1111/pbi.12532 (PMC5067624; doi:10.1111/pbi.12532)
Supplement: Supplementary file 2 — Table S1 Deletiontyping of the radiation hybrid (RH) lines used for bulk segregant analysis, modified from Michalak et al. (2013). Table S2 Contigs (Ctg) used for RH mapping, their gene content and orthologous relationships. Table S3 Contigs with pseudogenes. Table S4 Sequences of primers, probes, and adapters used in this study. Table S5 PDB Sum prediction of turning amino acids for transmembranes of the rhomboid protein (chr. 1D‐type). The amino acid (R) caused by the non‐synonymous HSV is indicated in bold. Table S6 NCBI protein entries identified across the plant kingdom as similar to scs ae and their reported alignment value to scs ae (Triticum chr 1D). [file PBI-14-1716-s002.docx]

**Fast-forward genetics by radiation hybrids to saturate the locus regulating nuclear-cytoplasmic compatibility in *Triticum***

Filippo M. Bassi, Farhad Ghavami, Matthew J. Hayden, Yi Wang, Kerrie L. Forrest, Stephan Kong, Rhoderissa Dizon, Monika K. Michalak de Jimenez, Steven W. Meinhardt, Mohamed Mergoum, Yong Q. Gu, and Shahryar F. Kianian

**Supplementary Information**

## BulkSeq by genome complexity reduction

Informative RH lines were selected from the population employed by Michalak et al., 2013 for fine mapping of the *scs^ae^* locus. Six RH_1_ lines were phenotypically selected for each of the ∆*scs^ae^* and *scs^ae^* (wild-type) phenotypes (SI Table 1). Genomic DNA was extracted from each line using a modified phenol:chloroform method (Hossain et al., 2004b), then equilibrated to 100 ng/µl by means of a NanoDrop spectrophotometer (Thermo Scientific, DE). Twenty µl of each DNA equilibration was pooled to create a negative bulk (bulk^NEG^ = ∆*scs^ae^*) and a positive bulk (bulk^POS^ = *scs^ae^*). Hence, DNA of the bulk^POS^ represented the whole genome [13” + 1A’ + 1D’] without any unmasked deletions, while the DNA of the bulk^NEG^ represented the entire genome, with the exclusion of the *scs^ae^* region, which was deleted in all the mutant lines. Two µg of each DNA pool was digested using 10 U *Pst*I (or *Aat*II) in 1x NEB Buffer 3 (New England Biolabs), 1x BSA, 1 mM ATP and 100 nM PstI_PE2 (or AatII_PE2) adapter (SI Table 4) in a final volume of 50 µl. The reactions were incubated at 37°C for 1 h before adding 6 Weiss units of T4 DNA ligase (New England Biolabs) for ligation of the adapter to the cleaved ends of the genomic DNA. The reactions were incubated at 37°C for a further 2 h, then at 65°C for 20 min to inactivate the enzymes. *Pst*I and *Aat*II are methylation-sensitive restriction endonucleases that cleave only non-methylated DNA. Both enzymes have been shown to preferentially cleave on low-copy genes-rich regions of the wheat genome (Wenzl et al., 2010). The PE2 adapter-ligated products were purified using a QIAquick PCR purification column (Qiagen), and physically sheared by Covaris fragmentation (1x TE, 5% duty cycle, intensity of 3200 cycles per burst, 80 sec) in a total volume of 120 µL. The randomly fragmented products were again purified using a QIAquick PCR purification column and end-polished using the Next End Repair kit (New England Biolabs). Following purification using a QIAquick PCR purification column, the products were dA-tailed in 1x NEB Buffer 2, 0.2 mM dATP and 15 U Klenow fragment (3’->5’ exo^-^) at 37°C for 30 min. The Kelnow enzyme was inactivated by heating at 65°C for 20 min. TA_PE1 adapter was ligated to the randomly sheared ends of the genomic DNA fragments by adding to the existing reaction 1x NEB Buffer 2, 1 mM ATP, 1x BSA, 0.8 µM TA_PE1 adapter (SI Table 4) and 6 Weiss units of T4 DNA ligase (New England Biolabs) to give a final volume of 65 µl. The ligation reaction was incubated at 12°C overnight. The ligation products were purified using a QIAquick PCR purification column, size-selected by gel excision (1.5% 1x TAE agarose gel, 475 ± 50 bp gel cut) and purified using a QIAquick gel extraction kit (Qiagen).The DNA fragments were selectively enriched by PCR for those having a PE1 adapter at one end and a PE2 adapter at the other end. PCR was performed in 2x Phusion HF PCR buffer (New England Biolabs), 0.2 mM dNTP, 1/5000 SYBR green I mix, 1.5 U Phusion DNA polymerase (New England Biolabs), 200 nM each mpxPE PCR primer (SI Table 4) and 2 µl of purified size-selected genomic DNA fragments in a final volume of 30 µl. Following initial incubation at 98°C for 30 s, PCR was performed for 16 cycles of 98°C for 10 s, 65°C for 20 s and 72°C for 30 s in a Stratagene MX305 qPCR thermocycler. The PCR products were SPRI (Argencourt) purified according to the manufacturer’s instructions using a 1:1 bead to sample volume ratio. The resulting complexity reduced *Pst*I and *Aat*II libraries for each of bulk^NEG^ and bulk^POS^ were quantified by KAPA titration (KAPA Biosystems) according to manufacturer’s instruction, and sequenced in two lanes of HiSEQ2000 using 100-bp pair-end sequencing chemistry (Illumina). Due to unidirectional adapter ligation, the PE1 read corresponds to the end of the DNA fragments with a random physical shear, whereas the PE2 read corresponds to DNA ends having a restriction enzyme cleavage site. The PE2 reads from bulk^POS^ and bulk^NEG^ samples were *de-novo* assembled using Stacks software (Catchen et al., 2011) to generate reference contig sequences corresponding to *Pst*I (or *Aat*II) loci in the wheat genome. A minimum of 3 reads and 98% sequence identity was required for contig formation. Next, PE2 reads from bulk^POS^ and bulk^NEG^ were aligned separately to the PE2 contigs using the BWA algorithm allowing for no mismatches (Li and Durbin, 2009). Bulk^POS^-specific sequences were identified as contigs that had high read coverage in the bulk^POS^ sample and no coverage in the bulk^NEG^ sample. To find an appropriate false-discovery cut-off to account for sampling error, the maximum read count for bulk^NEG^-specific reads was identified. As there is no biological basis for having bulk^NEG^-specific reads, all bulk^NEG^-specific reads were expected to be the result of incomplete coverage of the opposite bulk. The false discovery cut-off was found to be 58X coverage for the *Pst*I library and 269X for the *Aat*II library. To be conservative and minimize the false-discovery rate, the minimum read coverage for declaring a bulk^POS^-specific contig was set at 100 for the *Pst*I library and 500 for the *Aat*II library. PE1 reads corresponding to the opposite end of PE2 cleavage sites specific to bulk^POS^ were retrieved using custom perl scripts and *de novo* assembled using CAP3 (Huang and Madan, 1999) at 98% sequence identity.

## Assembly of contigs and annotation

The bulk^POS^-specific reads were run through a pipeline that employs the 53X survey sequence of the D-genome progenitor *A. tauschii* to extend the input sequences. Briefly, the bulk^POS^-specific reads are used as input to query the *A. tauschii* database and identify 100% identity fragments. These fragments are then assembled onto the input sequence, and the procedure is repeated using the newly assembled fragment. The procedure continues until a repetitive sequence is encountered, at which point the software stops. Each input sequence is extended independently from the others. The extended sequences were then assembled into contigs using the SeqMan Pro DNAStar software (Lasergene, Madison, WI), maintaining the default parameters except that FASTA sequences were used as input, and that any contig with at least 1 sequence of any length was accepted as output. These extended and assembled contigs were then annotated employing rice as model in the SNAP Gene application available on the discovery environment of the ‘iPlant Collaborative’ ([http://www.iplantcollaborative.org](http://www.iplantcollaborative.org/discover/discovery-environment); Goff et al., 2011). The annotated coding sequences were then run on the ‘Wheat Zapper’ application at E= e^-10^ (available at: <http://wge.ndsu.nodak.edu/wheatzapper/>; Alnemer et al., 2013) to identify any orthologus gene by synteny conservation with three model species (*Oryza sativa* L., *Sorghum bicolor* Moench, and *Brachypodium distachyon* eauv.). The contigs containing genes were then aligned to the wheat tentative consensus (TC) EST sequences (<http://compbio.dfci.harvard.edu/tgi/plant.html>; Quackenbush et al., 2001) provided by the ‘Wheat Zapper’ through multiple blastn alignment (available at: http://blast.ncbi.nlm.nih.gov/). Those ORF that provided less than 30% coverage of the TC sequence were considered as pseudogenes, and were excluded from further analysis. For the remaining ORF representing complete genes, the position of their exons/introns junctions and the non-genic spaces was accurately annotated for each contig by means of desktop analysis using the ‘Blast Align’ tool. Primers were designed on non-coding portions using the ‘Primer-blast’ application (Ye et al., 2012). These primers were defined NDCtg and used to confirm the quality of the assembly. Failure to amplify the DNA of LDN 1D(1A) was considered as a possible error in the assembly. The contigs were ordered along the length of a RH map to generate a continuous gapped sequence of the *scs^ae^* region by genotyping the 644 RH_1_ lines with the 1D-specific NDCtg. BAC Contig 30 was identified as containing the Xndsu297 markers by means of markers alignment. The minimal tiling path (MTP) of the contig contained 21 overlapping BAC clones. To sequence MTP BAC clones, BAC DNA from every 7 overlapping clones were pooled with equal amount and 1ug of pooled DNA was sheared into fragments with an average size around 850 Kb. The sheared DNA was indexed and used to construct the sequencing library using KAPA HTP Library Preparation Kit Illumina platforms following the manufacturer’s instructions. The prepared libraries were sequenced on the Illumina MiSeq machine using Illumina MiSeq Reagent Kit V3. About 100X coverage of Illumina reads with 300 bp from both ends were used to assemble overlapping BAC clones with SOAPdenovo V2.01, resulting in N50 contig size around 110 kb. BAC ends were generated for each clones and used to order and orient sequence contigs along MTP clones. The assembled sequence was further validated and improved using the genome mapping in nanochannel arrays technology as described previously (Hastile et al. 2013).

**References**

Catchen, J.M., Amores, A., Hohenlohe, P., Cresko, W. and Postlethwait, J.H. (2011). Stacks: building and genotyping loci de novo from short read sequences. *G3: Genes, Genomes, Genetics*, **1**, 171-182.

Hastile, A.R. *et al.* (2013) Rapid genome mapping in nanochannel for highly complete and accurate de novo sequence assembly of the complex *Aegilops tauschii* genome. *PLOS One,* **8,** e55864.

Hossain, K.G., Riera-Lizarazu, O., Kalavacharla, V., Vales, M.I., Rust, J.L., Maan, S.S. and Kianian, S.F. (2004b). Molecular cytogenetic characterization of an alloplasmic durum wheat line with a portion of chromosome 1D of Triticum aestivum carrying the scsae gene. *Genome*, **47**, 206-214.

Huang, X. and Madan, A. (1999). CAP3: A DNA sequence assembly program. *Genome Res*,. **9**, 868-877.

Li, H. and Durbin, R. (2009). Fast and accurate short read alignment with Burrows-Wheeler Transform. *Bioinformatics*, **25**, 1754-1760.

Quackenbush, J. *et al.* (2001). The TIGR gene indices: analysis of gene transcript sequences in highly sampled eukaryotic species. *Nucl. Acids Res.,* **29**, 159-164.

Wenzl, P. *et al.* (2010). Isolated chromosomes as a new and efficient source of DArT markers for the saturation of genetic maps. *Theor. Appl. Genet.,* **121**, 465–474.

Ye, J., Coulouris, G., Zaretskaya, I., Cutcutache, I., Rozen, S. and Madden, T.L. (2012). Primer-BLAST: A tool to design target-specific primers for polymerase chain reaction. *BMC Bioinformatics,* **13**, 134.

**SI Table 1.** Deletiontyping of the radiation hybrid (RH) lines used for bulk segregant analysis, modified from Michalak et al. (2013)

| RH lines | cR | 67 | 3057 | 3058 | 3027 | 3079 | 3064 |  | Bulk ^NEG^ | Bulk ^POS^ |
| --- | --- | --- | --- | --- | --- | --- | --- | --- | --- | --- |
| Phenotype ^a^ |  | 11:13 | 26:24 | 25:25 | 24:28 | 54:57 | 34:33 |  | 174:180 | 232:90 |
| Ratio |  | 1:1 | 1:1 | 1:1 | 1:1 | 1:1 | 1:1 |  | 1:1 | 3:1 |
|  |  |  |  |  |  |  |  |  |  |  |
| *Xndsu211* | 0 | 1 | 0 | 0 | 1 | 1 | 1 |  | 1 | 1 |
| *Xndsu212* | 10 | 0 | 0 | 0 | 0 | 1 | 1 |  | 1 | 1 |
| *Xndsu13* | 10.3 | 0 | 0 | 0 | 0 | 1 | 1 |  | 1 | 1 |
| *Xndsu222* | 10.5 | 0 | 0 | 0 | 0 | 1 | 1 |  | 1 | 1 |
| *Xndsu223* | 10.5 | 0 | 0 | 0 | 0 | 1 | 1 |  | 1 | 1 |
| *XNDCtg235A* | 10.8 | 0 | 0 | 0 | 0 | 0 | 0 |  | 0 | 1 |
| *XNDCtg6C* | 10.8 | 0 | 0 | 0 | 0 | 0 | 0 |  | 0 | 1 |
| *XNDCtg6A* | 10.8 | 0 | 0 | 0 | 0 | 0 | 0 |  | 0 | 1 |
| *XNDCtg49A* | 10.9 | 0 | 0 | 0 | 0 | 0 | 0 |  | 0 | 1 |
| *XNDCtg49B* | 10.9 | 0 | 0 | 0 | 0 | 0 | 0 |  | 0 | 1 |
| *XNDCtg108A* | 11.2 | 0 | 0 | 0 | 0 | 0 | 0 |  | 0 | 1 |
| *XNDCtg5B* | 11.2 | 0 | 0 | 0 | 0 | 0 | 0 |  | 0 | 1 |
| *Xndsu297* | 11.5 | 0 | 0 | 0 | 0 | 0 | 0 |  | 0 | 1 |
| *XScs^ae^* | 11.5 | 0 | 0 | 0 | 0 | 0 | 0 |  | 0 | 1 |
| *Xndsu296* | 12.2 | 0 | 0 | 0 | 0 | 0 | 0 |  | 0 | 1 |
| *Xndsu225* | 12.2 | 0 | 0 | 0 | 0 | 0 | 0 |  | 0 | 1 |
| *XNDCtg478A* | 13.5 | 0 | 0 | 0 | 0 | 0 | 0 |  | 0 | 1 |
| *Xndsu19* | 13.5 | 0 | 0 | 0 | 0 | 0 | 0 |  | 0 | 1 |
| *XNDCtg478E* | 13.5 | 0 | 0 | 0 | 0 | 0 | 0 |  | 0 | 1 |
| *XNDCtg478C* | 13.5 | 0 | 0 | 0 | 0 | 0 | 0 |  | 0 | 1 |
| *Xndsu18* | 13.8 | 0 | 0 | 0 | 0 | 0 | 0 |  | 0 | 1 |
| *Xndsu294* | 14.2 | 0 | 0 | 0 | 0 | 0 | 0 |  | 0 | 1 |
| *XNDCtg77C* | 14.2 | 0 | 0 | 0 | 0 | 0 | 0 |  | 0 | 1 |
| *XNDCtg77B* | 14.5 | 0 | 0 | 0 | 0 | 0 | 0 |  | 0 | 1 |
| *XNDCtg126C* | 14.8 | 0 | 0 | 0 | 0 | 0 | 0 |  | 0 | 1 |
| *Xndsu295* | 14.8 | 0 | 0 | 0 | 0 | 0 | 0 |  | 0 | 1 |
| *Xndsu291* | 14.8 | 0 | 0 | 0 | 0 | 0 | 0 |  | 0 | 1 |
| *Xndsu20* | 15.3 | 0 | 0 | 0 | 0 | 0 | 1 |  | 1 | 1 |
| *Xndsu21* | 15.7 | 0 | 0 | 0 | 0 | 1 | 1 |  | 1 | 1 |
| *Xndsu298* | 15.7 | 0 | 0 | 0 | 0 | 1 | 1 |  | 1 | 1 |
| *Xndsu224* | 15.7 | 0 | 0 | 0 | 0 | 1 | 1 |  | 1 | 1 |
| *Xndsu31* | 15.8 | 0 | 0 | 0 | 0 | 1 | 1 |  | 1 | 1 |
| *Xndsu3* | 15.8 | 0 | 0 | 0 | 0 | 1 | 1 |  | 1 | 1 |
| *Xndsu226* | 16.1 | 0 | 0 | 0 | 0 | 1 | 1 |  | 1 | 1 |
|  |  |  |  |  |  |  |  |  |  |  |

^a^ Measured as ratio between number of plump to shriveled seeds after testcrossing.

**SI Table 2**. Contigs (Ctg) used for RH mapping, their gene content and orthologous relationships

| Gene ID | Wheat EST | Rice gene | Brachy gene | Sorghum gene | Pfam description |
| --- | --- | --- | --- | --- | --- |
| Ctg5.1 | CA664118 | Os10g37760 | Bd3g31500 | Sb01g031210 | OsRhmbd17 - Rhomboid homologue |
| Ctg5.2 | TC421065 | Os03g02540 | Bd3g31500 | Sb01g049510 | Proteasome subunit |
| Ctg6.1 | TC391886 | Os10g36340 | Bd3g30370 | Sb01g017640 | Expressed protein |
| Ctg6.2 | CJ727521 | Os10g36250 | Bd3g30360 | Sb01g017670 | Tetratricopeptide repeat |
| Ctg6.3 | TC411602 | Os10g36210 | Bd3g30350 | Sb01g039010 | Valyl.tRNA synthetase |
| Ctg6.4 | BE425613 | Os10g36190 | Bd3g30330 | Sb01g017690 | PPR repeat domain containing protein |
| Ctg6.5 | TC373225 | Os10g36200 | Bd3g30340 | Sb01g017680 | Heavy metal.associated domain containing protein |
| Ctg6.6 | CJ632301 | . | . | . | . |
| Ctg49.1 | TC373225 | . | . | . | . |
| Ctg49.2 | CA677620 | Os10g35680 | Bd3g30050 | Sb01g018040 | Acetyltransferase GNAT family |
| Ctg49.3 | TC423735 | Os10g35670 | Bd3g30040 | Sb01g018050 | Zinc finger RING-type |
| Ctg49.4 | TC373225 | . | . | . | . |
| Ctg77.1 | CJ618747 | Os04g47500 | Bd5g05150 | Sb10g008140 | Transposon protein Pong sub-class |
| Ctg77.2 | DR740874 | Os10g36580 | Bd3g30730 | Sb01g017440 | UP-9A |
| Ctg77.3 | TC424578 | . | Bd3g30740 | Sb01g017450 | Expressed protein |
| Ctg77.4 | CA634829 | Os10g36550 | Bd3g30750 | Sb01g017460 | CRP3 - Cysteine-rich family protein precursor |
| Ctg108.1 | BQ239693 | Os10g35800 | Bd3g30140 | Sb01g017900 | Expressed protein |
| Ctg108.2 | TC445720 | Os10g35810 | Bd3g30150 | Sb01g017890 | Thylakoid lumenal protein |
| Ctg108.3 | TC423679 | Os12g39330 | Bd4g29010 | Sb01g014810 | AP2 domain containing protein |
| Ctg123.1 | TC424578 | . | . | . | . |
| Ctg123.2 | TC373225 | Os03g02260 | Bd3g57450 | Sb01g017050 | DnaK family protein |
| Ctg126.1 | . | . | . | . | . |
| Ctg179.1 | CA727403 | Os03g02380 | Bd3g31140 | Sb01g016960 | Major facilitator superfamily domain-containing protein 5 |
| Ctg179.3 | CA485571 | Os03g02390 | Bd3g31150 | Sb01g016950 | Mitochondrial inner membrane translocase Tim17 |
| Ctg235.1 | TC426369 | Os10g36060 | Bd3g30280 | Sb01g017720 | Protein transport protein-related |
| Ctg235.2 | CD373934 | Os10g36420 | Bd3g30410 | Sb01g017560 | YABBY domain containing protein |
| Ctg235.3 | TC391117 | Os10g36420 | Bd3g30410 | Sb01g017560 | YABBY domain containing protein |
| Ctg478.1 | TC391166 | Os10g37740 | Bd3g31480 | Sb01g031220 | CGMC_GSK.9 . CGMC MAPK GSK3 kinases |
| Ctg478.2 | CA502654 | Os10g37730 | Bd3g31460 | Sb01g031230 | pollen ankyrin |
| Ctg478.3 | CK193445 | Os10g37720 | Bd3g31450 | Sb01g031240 | hydrolase alpha/beta fold family protein |
| Ctg478.4 | TC438177 | Os10g37710 | Bd3g31440 | Sb01g031250 | hydrolase alpha/beta fold family protein |
| Ctg478.5 | CF134152 | Os10g37710 | Bd3g31440 | Sb01g031240 | hydrolase alpha/beta fold family protein |
| Ctg478.6 | CJ632301 | . | . | . |  |
| Ctg506.1 | TC388875 | Os01g39260 | Bd1g02250 | Sb03g025820 | OsFtsH5 FtsH protease homologue of AtFtsH4 |
|  |  |  |  |  |  |

**SI Table 3**. Contigs with pseudogenes

| Contig ID | Wheat  EST | Orthologus genes | | | Pfam description |
| --- | --- | --- | --- | --- | --- |
|  |  | Rice gene | *Brachypodium* | Sorghum |  |
| Ctg10.1 | TC431558 | Os04g40990 | Bd5g13940 |  |  |
| Ctg11.1 | EB511847 | Os10g37899 | Bd3g31570 | Sb06g020720 | malate synthase glyoxysomal |
| Ctg72.1 | TC375292 | Os02g09670 | Bd3g06570 | . | serine hydroxymethyltransferase mitochondrial precursor |
| Ctg130.1 | CV770052 | Os09g23560 | . | Sb03g013170 | S-adenosylmethionine synthetase |
| Ctg176.1 | CA699070 | Os12g22030 | Bd4g08100 | Sb03g025720 | carbamoyl-phosphate synthase large chain |
| Ctg199.1 | TC397718 | Os09g12590 | Bd1g14150 | . | 2-oxoglutarate dehydrogenase E1 mitochondrial precursor |
| Ctg199.2 | CV766600 | Os01g22010 | Bd2g12160 | Sb02g023310 | succinate dehydrogenase and fumarate reductase iron-sulfu |
| Ctg200.1 | TC398829 | Os02g21750 | Bd3g12630 | Sb0221s002050 | protein |
| Ctg204.2 | CK210080 | Os03g63490 | . | Sb06g017820 | protein |
| Ctg231.1 | TC454342 | Os02g49720 | . | . | L-aspartate oxidase 1 |
| Ctg240.1 | TC412091 | Os03g04410 | . | . | aldehyde dehydrogenase |
| Ctg242.1 | TC417842 | Os03g31300 | Bd5g08920 | . | elongation factor |
| Ctg281.2 | TC373948 | Os01g38970 | Bd2g41480 | . | MDR-like ABC transporter |
| Ctg471.2 | TC368579 | Os04g16830 | Bd5g23820 | . | MDR-like ABC transporter |
| Ctg485.1 | TC461358 | Os02g38210 | Bd3g47690 | . | MDR-like ABC transporter |
| Ctg498.1 | TC383944 | Os02g04170 | Bd3g03080 |  |  |
|  |  |  |  |  |  |

**SI Table 4**. Sequences of primers, probes, and adapters used in this study

| Marker | Primer forward | Primer reverse |
| --- | --- | --- |
| NDCtg49A | CGGGAAGGAATCTACCTCGC | ATTTTCCCCCGCTTCTTCCC |
| NDCtg49B | CCACACCACACACATTTGCC | GGGTTCTTGCGATCTACCCG |
| NDCtg235A | GGAAGGATTGTGTGTGTAGGC | ATCTGGCCGAAGTTACACGG |
| NDCtg6A | TGTTTGTGCTTCTCATTTCTAGGC | AGATGCAACCAAATTCAGATAGC |
| NDCtg6C | GATTTTTCCGATTCGGGCGG | AAAACTTTCGCCAGCTTCGC |
| NDCtg478A | CTTCGACTCCATCACCAGGG | GAGCTGACACAGAGGATGGG |
| NDCtg478C | CTTACAGGCAACCTCCAGGG | CACTTGTCACCCATCTCCCC |
| NDCtg478E | TTCGCTTCTTTTTGCTCGCC | TCACTGGTGTGCATCTTCCC |
| NDCtg77C | AGCGGGGTTAGTACAATGGC | GCTCCCACTTCATCCTACGG |
| NDCtg77B | CACCAGCAAAGCATACTGCC | GGATCTCCCCTTTTTGGGGG |
| NDCtg108A | AGGTCTCTTTTCTTCGCCCC | ACAGATACACACACCAGCCG |
| NDCtg5B | GTTTCTATAAACTTAAGAAGGGGGC | TGTGCTATTGGAGATTTCGGG |
| NDCtg506C | GTAGGTGCATCTCGTGTGCG | GCAGGAAGAAGGTCACACCC |
| NDCtg506A | ATGAGATCGATGCCGTTGGC | CTACCATCGACACCACACGC |
| NDCtg126C | GTCTGACTCCACAAGCACCC | AAGAGGTGTTGACAGCAGCG |
| RHBD1 | AAACCGCGTCCACCCCACGC | CCCGAGGAGCGGGTTCTGGC |
| RHBD2 | TGGGTGCCGTGGATCGTGCC | GCAAGCAGGTGGACGACGCC |
| RHBD3 | CTGCATCTGGCTCCACGCCG | AGCGTCACCAGCGCCGCC |
| RHBD4 | CCTCCGGTGCGCTCTTCGGG | CGTTGCCGTTCACCCCTCGG |
| RHBD5 | AACTTCGCGCACATCGGGGG | GCGCATCAAAATGCCTGTGCG |
| RHBD6 | GCTGGCGGTTGGGATGGCG | TCATTAACACTTGCTTTCGGTGCGG |
| NDRT20 | GAGAGTCGGCGTCATCTACC | CACGAACAGCAGCGTCAC |
| NDRT20a Probe | [FAM] agagacaacatctccgtgggcgc [BHQ1] | |
| NDRT20b Probe | [TET] aaagacaacatctccgtgggcgc [BHQ1] | |
| Actin | GGCAACATTGTTCTCAGTGGTGGT | TCCTTTCAGGAGGAGCAACAACCT |
| *Pst*I.adp1(+)PE2 | C*CGTGACTGGAGTTCAGACGTGTGCTCTTCCGATCTATGCA | |
| *Pst*I.adp1(-)PE2 | 5Phos/TAGATCGGAAGAGCACACGTCTGAACTCCAGTCAC | |
| *Aat*II.adp1(+)PE2 | C*CGTGACTGGAGTTCAGACGTGTGCTCTTCCGATCTAACGT | |
| *Aat*II.adp1(-)PE2 | 5Phos/TAGATCGGAAGAGCACACGTCTGAACTCCAGTCAC | |
| TA_PE1_adp(+) | ACACTCTTTCCCTACACGACGCTCTTCCGATC*T | |
| TA_PE1_adp1(-) | 5Phos/GATCGGAAGAGCGTCGTGTAGGGAAAGAGTGT | |
| PE1 PCR primer | A*ATGATACGGCGACCACCGAGATCTACACTCTTTCCCTACACGAC | |
| PE2 PCR primer | C*AAGCAGAAGACGGCATACGAGATNNNNNNGTGACTGGAGTTCAGACGTGT | |
| *Pst*I.adp1(+)PE2 | C*CGTGACTGGAGTTCAGACGTGTGCTCTTCCGATCTATGCA | |
| *Pst*I.adp1(-)PE2 | 5Phos/TAGATCGGAAGAGCACACGTCTGAACTCCAGTCAC | |
| *Aat*II.adp1(+)PE2 | C*CGTGACTGGAGTTCAGACGTGTGCTCTTCCGATCTAACGT | |
| *Aat*II.adp1(-)PE2 | 5Phos/TAGATCGGAAGAGCACACGTCTGAACTCCAGTCAC | |

Adp, adapter; PE1 and PE2, pair end 1 and 2; NNNNNN, corresponds to a unique 6-bp barcode sequences for sample tracking of 100-bp pair-end reads generated on the HiSEQ2000 sequencer.

**SI Table 5**. PDB Sum prediction of turning amino acids for transmembranes of the rhomboid protein (chr. 1D-type). The amino acid (R) caused by the non-synonymous HSV is indicated in bold

|  |  |  |  | Turn |  |  |
| --- | --- | --- | --- | --- | --- | --- |
| Turn |  | Sequence |  | type |  | H-bond |
|  | | | | | | |
| Asp103-Val106 |  | DKVV |  | IV |  |  |
| Arg170-Ile173 |  | **R**DNI |  | IV |  |  |
| Asp171-Ser174 |  | DNIS |  | IV |  |  |
| Ala177-Ala180 |  | ASGA |  | IV |  | Yes |

**SI Table 6**. NCBI protein entries identified across the plant kingdom as similar to *scs^ae^* and their reported alignment value to *scs^ae^* (*Triticum* chr 1D)

| Species | NCBI ID | Max score | Total score | Query cover | E value | Identity |
| --- | --- | --- | --- | --- | --- | --- |
| *Triticum* chr 1D | AGE14563.1 | 654 | 654 | 1.00 | 0 | 1.00 |
| *Triticum* chr 1B | AHL43431.1 | 631 | 631 | 1.00 | 0 | 0.96 |
| *Triticum* chr 1A | AHL43434.1 | 640 | 640 | 1.00 | 0 | 0.98 |
| *Hordeum vulgare* | BAJ89286.1 | 566 | 566 | 1.00 | 0 | 0.95 |
| *Oryza sativa* | EAY79245.1 | 495 | 495 | 0.96 | 1E-180 | 0.79 |
| *Zea mays* | XP_008659873.1 | 488 | 488 | 0.90 | 4E-178 | 0.83 |
| *Sorghum bicolor* | XP_002467632.1 | 486 | 486 | 1.00 | 5E-177 | 0.77 |
| *Brachypodium distachyon* | XP_003558972.1 | 440 | 440 | 0.99 | 6E-159 | 0.67 |
| *Populus trichocarpa* | XP_002304474.2 | 409 | 409 | 0.91 | 4E-147 | 0.66 |
| *Vitis vinifera* | XP_002279077.1 | 404 | 404 | 0.91 | 7E-145 | 0.67 |
| *Arabidopsis thaliana* | NP_176500.1 | 396 | 396 | 0.99 | 7E-142 | 0.61 |
| *Medicago truncatula* | ACJ84634.1 | 389 | 389 | 0.97 | 4E-139 | 0.61 |
|  |  |  |  |  |  |  |
